# Neuroprotection Comparison of Rosmarinic Acid and Carnosic Acid in Primary Cultures of Cerebellar Granule Neurons

**DOI:** 10.3390/molecules23112956

**Published:** 2018-11-13

**Authors:** Faten Taram, Elizabeth Ignowski, Nathan Duval, Daniel A. Linseman

**Affiliations:** 1 Department of Biological Sciences, University of Denver, 2199 S. University Blvd., Denver, CO 80208, USA; faten.taram@gmail.com (F.T.); lizignowski@gmail.com (E.I.); 2 Knoebel Institute for Healthy Aging, University of Denver, 2155 E. Wesley Ave., Denver, CO 80208, USA; nathan.duval@du.edu

**Keywords:** nutraceuticals, rosemary, neuronal apoptosis, antioxidants, polyphenols

## Abstract

Neurodegenerative disorders such as amyotrophic lateral sclerosis (ALS), Alzheimer’s disease, and Parkinson’s disease, are characterized by the progressive loss of neurons in specific regions of the brain and/or spinal cord. Neuronal cell loss typically occurs by either apoptotic or necrotic mechanisms. Oxidative stress and nitrosative stress, along with excitotoxicity and caspase activation, have all been implicated as major underlying causes of neuronal cell death. Diverse nutraceuticals (bioactive compounds found in common foods) have been shown to have neuroprotective effects in a variety of in vitro and in vivo disease models. In the current study, we compared the neuroprotective effects of two polyphenolic compounds, rosmarinic acid and carnosic acid, which are both found at substantial concentrations in the herb rosemary. The capacity of these compounds to rescue primary cultures of rat cerebellar granule neurons (CGNs) from a variety of stressors was investigated. Both polyphenols significantly reduced CGN death induced by the nitric oxide donor, sodium nitroprusside (nitrosative stress). Rosmarinic acid uniquely protected CGNs from glutamate-induced excitotoxicity, while only carnosic acid rescued CGNs from caspase-dependent apoptosis induced by removal of depolarizing extracellular potassium (5K apoptotic condition). Finally, we found that carnosic acid protects CGNs from 5K-induced apoptosis by activating a phosphatidylinositol 3-kinase (PI3K) pro-survival pathway. The shared and unique neuroprotective effects of these two compounds against diverse modes of neuronal cell death suggest that future preclinical studies should explore the potential complementary effects of these rosemary polyphenols on neurodegenerative disease progression.

## 1. Introduction

According to a 2015 United Nations report, the number of people over 60 is expected to double in the next 35 years to almost 2.1 billion people. As our population shifts to a larger proportion of elderly individuals, neurodegenerative disorders including amyotrophic lateral sclerosis (ALS), Parkinson’s disease (PD), and Alzheimer’s disease (AD) will continue to become more prevalent. Because most neurodegenerative diseases are primarily sporadic in nature, an effective method of treatment would be to prevent or slow the dying of neurons in affected brain regions. Inflammation, misfolded proteins, mitochondrial dysfunction, oxidative stress, nitrosative stress, apoptosis, and excitotoxicity are all considered major factors underlying the pathology of various neurodegenerative diseases [1,2]. Thus, therapeutic agents that target several of these disease mechanisms may be the most viable treatment options.

Since ancient times, plants have been used as natural treatments for acute and chronic disorders. Over the last few decades, natural compounds possessing medicinal benefits (i.e., nutraceuticals) have been proposed as promising treatment options for many diseases due to their intrinsic antioxidant abilities in scavenging reactive oxygen species (ROS) and reactive nitrogen species (RNS) [3,4]. The activities of many of these compounds, such as rosmarinic acid and carnosic acid, have been extensively investigated using in vitro and in vivo models [5]. Manufactured or commercially synthesized drugs sometimes display more activity than natural compounds. However, natural compounds often produce long term benefits and generally elicit fewer side effects [6]. Many nutraceuticals are classified as (poly)phenolic antioxidant compounds, like phenolic acids, flavonoids, tannins, and lignans [7,8].

**Figure 1:**
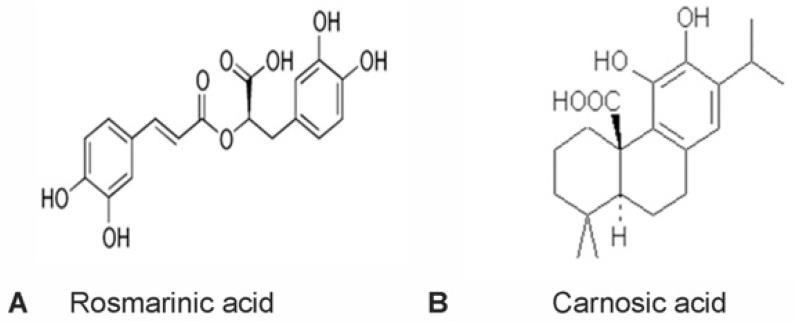
Structural comparison of rosmarinic acid (**A**) and carnosic acid (**B**).

The rosemary shrub (*Rosmrinus officinalis*) is a member of the mint family (*Lamiaceae*) found originally in the Mediterranean region, and now found abundantly throughout the world. Commonly used as an herbal spice in food, rosemary has long been used as an alternative therapy for different illnesses including headaches, inflammatory diseases, and stomach problems [9,10]. Rosemary has a significant intrinsic antioxidant activity due to its molecular components, such as rosmarinic acid and carnosic acid, and both compounds have demonstrated neuroprotective effects in different neurodegenerative diseases. The main constituent, rosmarinic acid (Figure 1A), is a naturally occurring hydroxylated polyphenol molecule found in several plant families, including *Lamiaceae*, *Boraginaceae*, and *Blechnaceae* [11,12]. It has been shown to have antioxidant, anti-inflammatory, anti-viral, anti-mutagenic, and anti-angiogenic capabilities [9,10,13,14,15,16,17]. Moreover, rosmarinic acid appears to be neuroprotective and has been demonstrated to induce neuroprotection from reactive glial cells in in vitro models of AD, spinal cord injury, and PD by inhibiting nitric oxide (NO) production [18,19,20]. Rosmarinic acid protected against memory deficits in ischemic mice and alleviated neurological symptoms in the G93A mutant hSOD1 mouse model of ALS [21,22,23].

Carnosic acid (Figure 1B), a polyphenolic compound also found in the rosemary herb, possesses many of the biological activities discussed above. The robust antioxidant activity of carnosic acid may be related to its ability to up-regulate endogenous free radical scavenging enzymes via activation of the Nrf2 transcriptional pathway [24,25]. In several in vitro models, carnosic acid has been shown to have protective effects against neurotoxicity by promoting pro-survival pathways including modulation of apoptosis, autophagy, and regulation of the parkin pathway [26,27]. Carnosic acid showed a neuroprotective effect both in vitro and in vivo in a mouse model of neurotoxicity and in a rat model of PD [28,29].

The purpose of this study was to directly compare the neuroprotective effects of rosmarinic acid and carnosic acid against cell death induced by nitrosative stress, excitotoxicity, and caspase activation in an in vitro model of primary rat cerebellar granule neuron (CGN) cultures. Apoptosis was measured morphologically by Hoechst staining to visualize nuclear size and morphology, in addition to bright field imaging or immunocytochemistry to visualize neuronal processes and the microtubule network, respectively. To further investigate the pro-survival pathway that carnosic acid activates to block caspase-dependent cell death, we used several different inhibitors, including wortmannin to inhibit PI3K, PD98059 to block MAPK (MEK1/2), and Akt inhibitor XIII to inhibit Akt-dependent pro-survival signaling. Our data demonstrate that rosmarinic acid and carnosic acid show overlapping and unique neuroprotective effects against diverse stressors that cause neuronal cell death. The unique neuroprotective effects of these two polyphenols has direct implications for designing future preclinical studies to evaluate the therapeutic potential of these compounds in neurodegenerative diseases.

## 2. Results

### 2.1. Carnosic Acid and Rosmarinic Acid Each Protect CGNs from Nitrosative Stress

**Figure 2:**
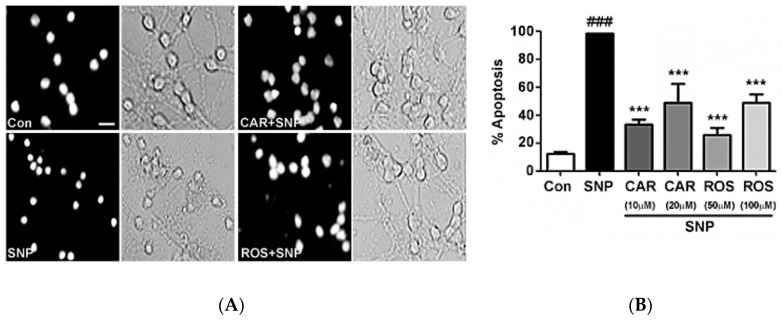
Rosmarinic acid and carnosic acid each protect cerebellar granule neurons (CGNs) from nitrosative stress. (**A**) CGNs were co-incubated for 24 h in serum-free culture medium containing 25 mM KCl (Control; Con) alone, or with rosmarinic acid (ROS; 50 μM) or carnosic acid (CAR; 10 μM) and sodium nitroprusside (SNP; 100 μM), or SNP alone. Following incubation, CGNs were fixed and stained with Hoechst dye to visualize the nuclei. Decolorized (black & white) panels are shown to emphasize nuclear morphology and gray panels show the bright field images of the same fields. Scale bar indicates 10 µm. (**B**) Quantitative assessment of cellular apoptosis for CGNs in untreated controls, SNP alone, and SNP plus ROS or CAR. Cells were quantified by counting as either living or apoptotic based on nuclear morphology, and the percentage of cells showing apoptotic nuclei (either condensed or fragmented morphology) was determined. Data are expressed as the means ± SEM, *n* = 4. ^###^ indicates *p* < 0.001 compared to control, *** indicates *p* < 0.001 compared to SNP alone as determined using one-way ANOVA with a post hoc Tukey’s test.

Rosmarinic acid and carnosic acid each protect CGNs from nitrosative stress induced by sodium nitroprusside (SNP), an NO donor, which results in the formation of RNS and cell death. The efficacy of rosmarinic acid and carnosic acid to protect CGNs against SNP-induced nitrosative stress was examined. By assessing the nuclear morphology and by examining the state of the neuronal processes under bright field imaging, we show cytotoxic effects of SNP on CGNs. When incubated with SNP, the nuclei become condensed and fragmented compared to the nuclei of untreated control cells (Figure 2A). Additionally, the neuronal cell bodies are drastically diminished in size and the neuronal processes are essentially destroyed by SNP (Figure 2A). These detrimental effects of RNS were substantially reduced by co-incubating the CGNs with either 50 µM rosmarinic acid or 10 µM carnosic acid (Figure 2A). Quantification of these results is shown in Figure 2B as the percentage of apoptotic cells counted using Hoechst staining to assess nuclear morphology. Rosmarinic acid significantly reduced apoptotic cell death at concentrations of 50 µM and 100 μM. Carnosic acid demonstrated a more potent protective effect from RNS at concentrations of only 10 µM and 20 µM. Interestingly, for both compounds, the protection observed was somewhat greater at the lower concentration tested. Of note, at concentrations of over 20 µM and 100 µM for carnosic acid and rosmarinic acid, respectively, the compounds were somewhat toxic on their own to CGNs (data not shown).

### 2.2. Rosmarinic Acid Uniquely Protects CGNs against Excitotoxicity

**Figure 3:**
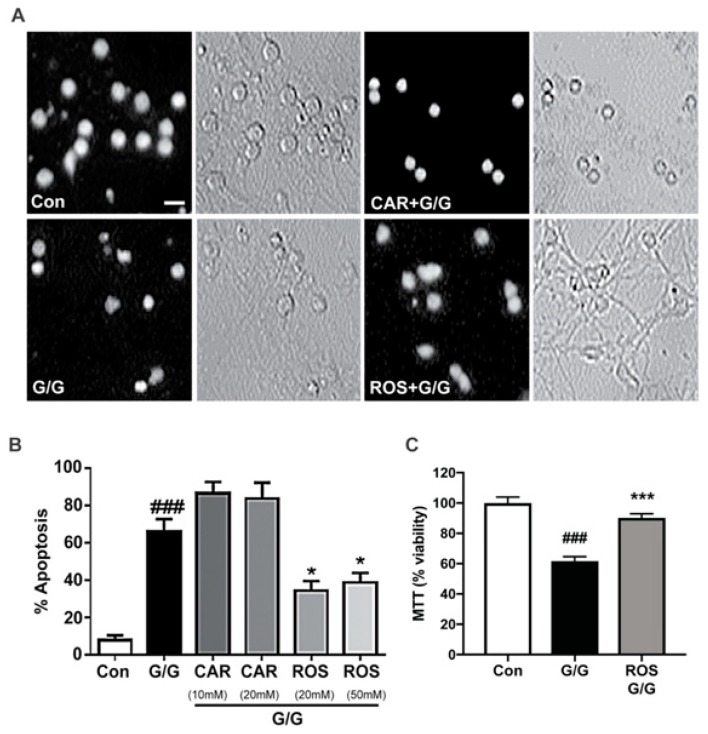
Rosmarinic acid, but not carnosic acid, protects CGNs from excitotoxicity. (**A**) CGNs were co-incubated for 24 h in serum-free culture medium containing 25 mM KCl (Control; Con) alone or with rosmarinic acid (ROS; 20 μM) or carnosic acid (CAR; 10 μM) and glutamate/glycine (G/G; 100 μM/10 μM final concentrations), or G/G alone. Following incubation, CGNs were fixed and stained with Hoechst dye to visualize the nuclei. Decolorized (black & white) panels are shown to emphasize nuclear morphology and the gray panels show the bright field images of the same fields. Scale bar indicates 10 µm. (**B**) Quantitative assessment of cellular apoptosis for CGNs in untreated controls, G/G alone, and G/G plus ROS or CAR. Cells were quantified as either living or apoptotic based on nuclear morphology, and the percentage of cells showing apoptotic nuclei (either condensed or fragmented morphology) was determined. (**C**) MTT cell viability assay was used to confirm the protective effects of ROS against G/G excitotoxicity. CGNs were co-incubated with 100 μM glutamate and 10 μM glycine (G/G), G/G + ROS and percentage of viability was calculated against control treatment. Data are expressed as the means ± SEM, *n* = 4. ^###^ indicates *p* < 0.001 compared to control, * indicates *p* < 0.05 compared to G/G alone, *** indicates *p* < 0.001 as determined using one-way ANOVA with a post hoc Tukey’s test.

Glutamate and its co-agonist glycine, cause excitotoxicity in CGN cultures by causing an influx of calcium. The molecules bind to ionotropic glutamate receptors, allowing calcium to enter facilitating depolarization of the cell. This increase in intracellular calcium can activate calpains, form ROS and RNS, and cause the neuron to fire more action potentials, resulting in further cell depolarization [30]. Induction of excitotoxicity by glutamate/glycine resulted in a marked increase in apoptotic CGNs and severe damage to neuronal processes (Figure 3A). Rosmarinic acid was protective against excitotoxicity; however, carnosic acid was not protective, and in fact appeared to exacerbate the effects of glutamate/glycine-induced excitotoxicity (Figure 3A). The percentage of apoptotic cells was determined for each treatment and the quantitative results are shown in Figure 3B. We performed a MTT assay of cell viability to confirm the protective effects of rosmarinic acid against glutamate/glycine-induced excitotoxicity. We show that CGNs incubated in glutamate/glycine resulted in significant cell death by excitotoxicity when compared to controls (Figure 3C). Furthermore, co-incubation of CGNs with glutamate/glycine and rosmarinic acid restores cell viability levels to control levels (Figure 3C). Our data confirm the protective effects of rosmarinic acid against glutamate/glycine-induced excitotoxicity. Rosmarinic acid significantly protected CGNs from excitotoxicity at 20 μM and 50 μM concentrations. In contrast, carnosic acid at concentrations of 10 μM and 20 μM enhanced CGN cell death in the presence of glutamate, though this effect did not reach statistical significance.

### 2.3. Carnosic Acid Uniquely Protects CGNs against Caspase-Dependent Apoptosis

**Figure 4:**
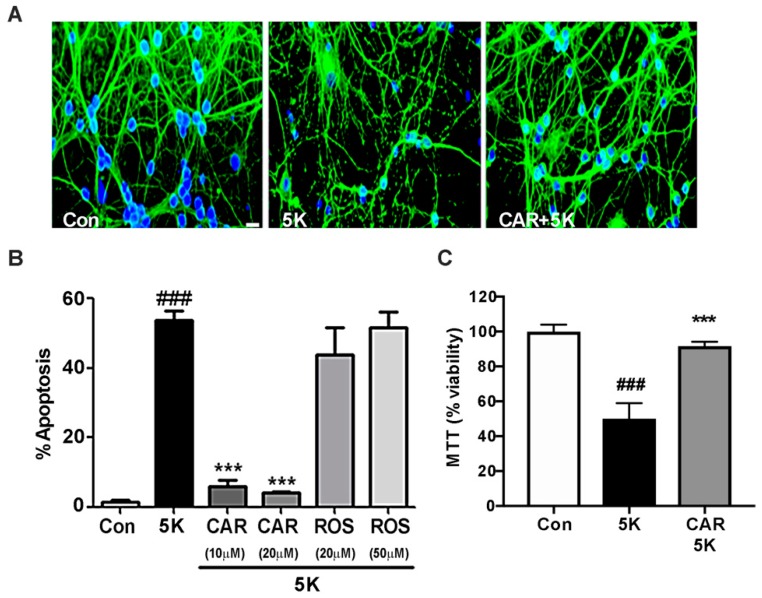
Carnosic acid, but not rosmarinic acid, protects CGNs from caspase-dependent apoptosis. (**A**) CGNs were co-incubated for 24 h in serum-free culture medium containing 25 mM KCl (Control; Con). Treated cells had 25 mM KCl medium replaced with a non-depolarizing, low potassium medium (5 mM KCl; 5K apoptotic medium) either alone or with carnosic acid (CAR; 20 μM). Following incubation, CGNs were fixed and stained with Hoechst (blue) and β-tubulin antibody (green) to visualize the nuclei and microtubule network, respectively. Scale bar indicates 10 µm. (**B**) Quantitative assessment of cellular apoptosis for CGNs in untreated controls, 5K apoptotic medium, and 5K plus ROS or CAR. Cells were quantified as either living or apoptotic based on nuclear morphology, and the percentage of cells showing apoptotic nuclei (either condensed or fragmented morphology) was determined. (**C**) MTT cell viability assay was used to confirm the protective effects of CAR against caspase-dependent apoptosis. CGNs were co-incubated with 5K, 5K + CAR and percentage of viability was calculated against control treatment. Data are expressed as the means ± SEM, *n* = 4. ^###^ indicates *p* < 0.001 compared to control, *** indicates *p* < 0.001 compared to 5K apoptotic condition alone as determined using one-way ANOVA with a post hoc Tukey’s test.

5K apoptotic medium is a non-depolarizing, low potassium medium that causes cellular stress and subsequent caspase activation in CGNs [31,32]. Caspases induce apoptotic cell death when activated. There are several pro-survival signaling pathways that can be inactivated by 5K medium to allow caspase activation resulting in cell death. Stimulation of these pathways under 5K conditions can therefore lead to cell survival and neuroprotection. The specific pathways we tested in subsequent experiments were the pro-survival PI3K/Akt pathway and PI3K-dependent activation of a MEK/ERK pathway. As expected, incubation in 5K medium induced apoptosis of CGNs as seen by the characteristic nuclear condensation and microtubule disruption (Figure 4A). Carnosic acid co-incubation significantly protected CGNs against 5K-induced apoptosis. These results were again quantified from Hoechst-stained images and the percentage of apoptotic cells was determined (Figure 4B). To confirm the protective effects of carnosic acid against caspase-dependent apoptosis, we performed an MTT assay of cell viability. CGNs incubated in 5K medium caused significant cell death and cell viability is restored when co-incubated with carnosic acid (Figure 4C). Our data confirm the protective effects of carnosic acid against low potassium induced caspase-dependent apoptosis. In contrast to the results described above for excitoxicity, rosmarinic acid did not display any discernible protective effect against caspase activation while carnosic acid nearly completely protected against the 5K apoptotic insult at 10 μM and 20 μM concentrations.

### 2.4. Carnosic Acid Protects CGNs from 5K-Induced Apoptosis through PI3K Activation, But Not Downstream Akt or MEK/ERK Signaling

**Figure 5:**
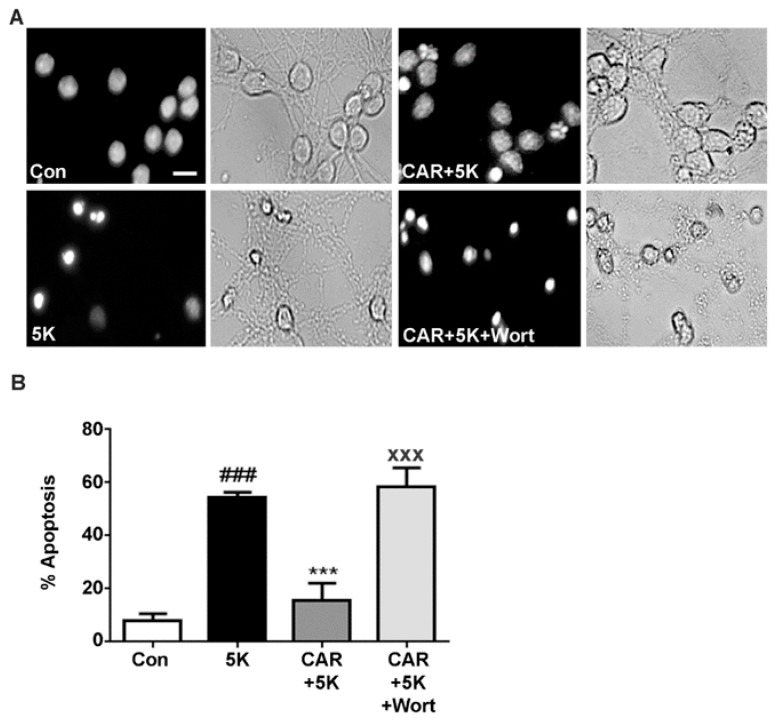
Carnosic acid protects CGNs from caspase-dependent apoptosis through the PI3K pro-survival pathway. (**A**) CGNs were incubated for 24 h in serum-free culture medium containing 25 mM KCl (Control; Con). Treated cells had the plating medium replaced with a non-depolarizing, low potassium medium (5 mM KCl; 5K apoptotic medium) either alone or co-treated with carnosic acid (CAR; 20 μM) or with CAR + wortmannin (Wort; 100 nM). Following incubation, CGNs were fixed and stained with Hoechst dye to visualize the nuclei. Decolorized (black & white) panels are shown to emphasize nuclear morphology and the gray panels show the bright field images of the same fields. Scale bar indicates 10 µm. (**B**) Quantitative assessment of cellular apoptosis for CGNs in untreated controls, 5K, 5K + CAR, and 5K + CAR + Wort. Cells were quantified by counting as either living or apoptotic based on nuclear morphology, and the percentage of cells showing apoptotic nuclei (either condensed or fragmented morphology) was determined. Data are expressed as the means ± SEM, *n* = 4. ^###^ indicates *p* < 0.001 compared to control, *** indicates *p* < 0.001 compared to 5K apoptotic medium, ^xxx^ indicates *p* < 0.001 compared to 5K + CAR, as determined using one-way ANOVA with a post hoc Tukey’s test.

Once it was determined that carnosic acid protected CGNs against 5K-induced, caspase-dependent apoptosis, we attempted to determine if these protective effects were due to activation of specific pro-survival pathways that would inhibit caspase activation. The PI3K pathway has known pro-survival effects. To investigate if carnosic acid acts through a PI3K-dependent mechanism, we used wortmannin, an irreversible inhibitor of PI3K. Bright field imaging and nuclear staining clearly showed that co-incubation with wortmannin counteracted the protective effects of carnosic acid against low-potassium cellular stress with 5K medium (Figure 5A). To quantify the induction of apoptosis in CGNs, we evaluated nuclear morphology which demonstrated that wortmannin effectively blocked the protective effect of carnosic acid seen in 5K apoptotic conditions (Figure 5B). This demonstrates that carnosic acid utilizes a PI3K-dependent mechanism of neuroprotection.

**Figure 6:**
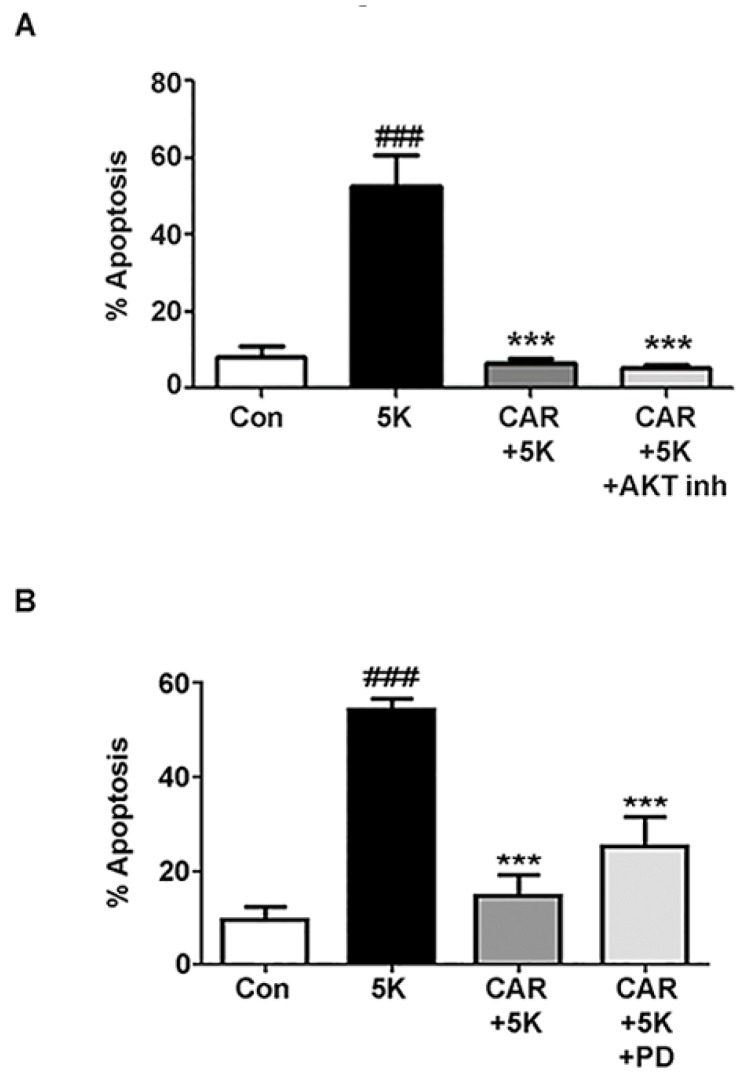
Carnosic acid protects CGNs from caspase-dependent apoptosis independently of the AKT or MEK/ERK pro-survival pathways. CGNs were incubated for 24 h in serum-free culture medium containing 25 mM KCl (Control; Con). Treated cells had the plating medium replaced with a non-depolarizing, low potassium medium (5 mM KCl; 5K apoptotic medium) either alone or co-treated with carnosic acid (CAR; 15 μM) alone or (**A**) CAR + AKT inhibitor (AKT inh; 10 μM) or (**B**) CAR + MEK inhibitor PD98059 (PD; 10 μM). Following incubation, CGNs were fixed and stained with Hoechst dye to visualize nuclei. (**A**,**B**) Quantitative assessment of cellular apoptosis for CGNs in untreated controls, 5K, 5K + CAR, 5K + CAR + AKT inh, and 5K + CAR + PD. Cells were quantified by counting as either living or apoptotic based on nuclear morphology, and the percentage of cells showing apoptotic nuclei (either condensed or fragmented morphology) was determined. Data are expressed as the means ± SEM, *n* = 4. ^###^ indicates *p* < 0.001 compared to control, *** indicates *p* < 0.001 compared to 5K apoptotic medium alone, as determined using one-way ANOVA with a post hoc Tukey’s test.

The pro-survival effects of PI3K are typically initiated through downstream phosphorylation and activation of the serine/threonine kinase Akt. Above we show carnosic acid exerts its neuroprotective effects in a PI3K-dependent manner. To determine if the neuroprotective effects are initiated through PI3K activation of Akt, we used the potent inhibitor, Akt Inhibitor XIII. Quantitative assessment of CGN apoptosis showed that surprisingly, the protective effect of carnosic acid was sustained even in the presence of the Akt inhibitor (Figure 6A). These results demonstrate that carnosic acid protects CGNs from 5K-induced apoptosis independently of the Akt pro-survival pathway.

Next, we investigated if the PI3K-dependent neuroprotective effect of carnosic acid was caused by a PI3K-dependent activation of Ras and subsequent downstream activation of the MAPK pathway. PI3K signaling through the Ras/MAPK pathway can promote cell survival. Using PD98059, a MEK1/2 inhibitor, we inhibited MAPK activation by binding and inactivating MEK1/2 and preventing downstream phosphorylation events [33]. Carnosic acid significantly protected CGNs from 5K-induced apoptosis even in the presence of PD98059 (Figure 6B). This result shows that carnosic acid protects CGNs from caspase-dependent apoptosis through a mechanism that is independent of the Ras/MAPK signaling pathway.

## 3. Discussion

Rosmarinic acid and carnosic acid have many bioactivities, including neuroprotective, antioxidant, and anti-inflammatory effects, that may make them viable therapeutic options to diminish the underlying pathophysiology of various neurodegenerative diseases. In the current study, we compared the neuroprotective effects of rosmarinic acid and carnosic acid against insults modeling nitrosative stress, excitotoxicity, and caspase-dependent apoptosis in cultured rat CGNs, a well-established in vitro model to examine neuronal cell death [34]. Moreover, we studied diverse mechanisms that may underlie the neuroprotective effects of carnosic acid against intrinsic apoptosis.

Our data show that rosmarinic acid and carnosic acid protect CGNs from an in vitro model of nitrosative stress induced by SNP. This compound is a NO donor which can promote the formation of toxic RNS [35]. The formation of RNS causes extensive cellular damage resulting in cell death [35]. The neuroprotective effects of rosmarinic acid against nitrosative stress in vivo likely involves its capacity to downregulate NF-ĸB and inhibit NO production from activated glial cells [36,37,38,39]. However, the effects observed in vitro in CGNs are likely due to rosmarinic acid directly scavenging NO. Moreover, both rosmarinic acid and carnosic acid share a structural motif; a catechol moiety that acts as a hydrogen donor to free radicals and uses oxygen as an electron acceptor [40]. Indeed, we have shown that polyphenols containing catechol moieties demonstrate robust neuroprotective effects against NO-induced toxicity [41,42]. Our results are also consistent with a previous study in which rosmarinic acid inhibited NO production in RAW2647 mouse macrophages [43]. In a similar manner, carnosic acid has been shown to inhibit NO production induced by LPS in microglial cells [44]. Thus, both rosmarinic acid and carnosic acid appear to mitigate nitrosative stress via two mechanisms. First, these compounds blunt NO production from macrophages and microglia. Second, they act as direct scavengers of RNS, including NO.

Excitotoxicity has been shown to play a role in these diseases and therefore, we tested these agents to determine their neuroprotective effects against glutamate/glycine-induced toxicity in CGNs [45]. To replicate excitotoxicity in vitro, CGNs were exposed to glutamate and its co-agonist glycine, to cause excitotoxicity by causing an influx of calcium. The molecules bind to ionotropic glutamate receptors, allowing calcium to enter, facilitating depolarization of the cell. One consequence of excitotoxicity is neuronal nitric oxide synthase (nNOS) activation and increase in NO, leading to nitrosative and oxidative stress [35]. We show that rosmarinic acid displays a neuroprotective effect against glutamate/glycine-induced excitotoxicity. This result has been substantiated in another study performed in human neuroblastoma cells [46]. Conversely, carnosic acid exacerbated the excitotoxic cell death in CGN cultures. The opposing effects of these compounds on excitotoxic cell death are striking and are not readily explained. Additional studies are necessary to define the mechanisms underlying this result.

In our study, we found that carnosic acid exacerbated the effects of excitotoxicity in CGNs. Other studies have shown it to antagonize intracellular calcium mobilization in leukocytes, and interestingly, it provided anti-excitotoxic effects in vivo and in vitro in HT-22 hippocampal cells [25,47]. The contrasting findings between our study and these prior reports may be due to differences in how the excitotoxic cell death is executed within the distinct cell types being tested. Regardless of these effects, carnosic acid did significantly protect CGNs against 5K apoptotic conditions, whereas rosmarinic acid did not.

To further characterize the protective effects of carnosic acid against intrinsic apoptosis, three different inhibitors were used to test the involvement of specific cell survival pathways. Inhibition of the PI3K pro-survival pathway with wortmannin blocked the protective effects of carnosic acid seen in 5K apoptotic medium. This demonstrated that the neuroprotective effects of this compound are dependent upon activation of a PI3K signaling pathway. Downstream of PI3K, two major pro-survival pathways can be activated, the Akt pathway and the Ras/MAPK pathway. When we inhibited Akt downstream of PI3K, carnosic acid still significantly protected CGNs against 5K-induced apoptosis suggesting that neuroprotection is not through Akt activation. Next, we examined the Ras/MAPK pathway by inhibiting MEK1/2 with PD98059. As with the Akt inhibitor, carnosic acid protected against 5K-induced apoptosis even in the presences of PD98059. This result shows that carnosic acid protects against intrinsic apoptosis independently of the Ras/MAPK pro-survival pathway.

We have tested two major pro-survival cell signaling pathways activated downstream of PI3K (Akt and Ras/MAPK) and shown that neither was essential to the protective effects of carnosic acid against intrinsic apoptosis, indicating that carnosic acid must protect CGNs through another PI3K-dependent pathway. One PI3K-dependent mechanism not tested, but potentially neuroprotective, is activation of the Nrf2 pathway. Previous studies reported that pretreating IMR-32 cells with wortmannin decreases Nrf2 nuclear translocation [48]. Furthermore, Satoh et al. have reported that carnosic acid activates Nrf2 in vivo and in vitro by activating the transcriptional antioxidant response element (ARE) modulating the cellular redox state [24,25]. Thus, the PI3K-dependent neuroprotection by carnosic acid against intrinsic apoptosis observed in CGNs may involve activation of the Nrf2 pathway. Another possibility is a PI3K-dependent activation of Rac GTPase and subsequent activation of p21-activated kinase (PAK), which we have previously shown to have pro-survival effects in CGNs [49,50,51]. Future studies will be necessary to examine if carnosic acid activates Rac/PAK pro-survival signaling in CGNs via PI3K and if this pathway is ultimately responsible for the protective effects of this polyphenol against intrinsic apoptosis.

In summary, rosmarinic acid and carnosic acid displayed both shared and unique neuroprotective effects when evaluated in cultured CGNs. Both compounds provided significant neuroprotection against the NO donor SNP, likely due to their shared catechol structural motif. On the other hand, only rosmarinic acid protected CGNs from excitotoxicity while carnosic acid uniquely protected CGNs from caspase-dependent apoptosis. Both of these compounds have been shown to have impressive antioxidant and neuroprotective effects and these effects nicely complement one another. Therefore, future preclinical studies should explore not only the individual therapeutic actions of these compounds, but also the potential complementary effects of these rosemary polyphenols on neurodegenerative disease progression.

## 4. Materials and Methods

### 4.1. CGN Culture

Cerebellar granule neurons (CGNs) were isolated as previously described [52] from seven-day-old Sprague Dawley rat pups. Cells were plated on poly-l-lysine coated six-well plates (35 mm-diameter), with a density of approximately 2 × 10^6^ cells/well in Basal Medium Eagle’s supplemented with 25 mM potassium chloride, 2 mM l-glutamate, 10% fetal bovine serum, and 2 mM penicillin-streptomycin (100 U/mL/100 µg/mL). Cytosine arabinoside (10 µM) was added to the culture medium 24 h after plating to inhibit the growth of non-neuronal cells. Cultures were ~95% pure for granule neurons. The resulting CGNs were then incubated at 37 °C in 10% CO_2_ for six to seven days in culture prior to experimentation. All animal manipulations were performed in accordance with a protocol approved by the University of Denver Institutional Animal Care and Use Committee.

### 4.2. Reagents

Sodium nitroprusside (SNP) and PD98059 were obtained from Calbiochem (San Diego, CA, USA). Carnosic acid ((4a*R*,10a*S*)-5,6-Dihydroxy-1,1-dimethyl-7-propan-2-yl-2,3,4,9,10,10 a-hexahydrophenanthrene-4a-carboxylic acid) and rosmarinic acid ((*R*)-*O*-(3,4-dihydroxy- cinnamoyl)-3-(3,4-dihydroxyphenyl) lactic acid, 3,4-Dihydroxycinnamic acid (*R*)-1-carboxy-2-(3,4-dihydroxyphenyl) ethyl ester) were obtained from AG Scientific (San Diego, CA, USA). Glutamic acid was purchased from MP biomedical (Santa Ana, CA, USA). Glycine, Hoechst, wortmannin, and monoclonal antibody to β-tubulin were obtained from Sigma Aldrich (St. Louis, MO, USA). Akt Inhibitor XIII was purchased from Calbiochem (San Diego, CA, USA).

### 4.3. Treatment Protocols

Treatment with glutamate/glycine and SNP:CGNs were co-treated with either carnosic acid or rosmarinic acid and the stressors SNP (100 μM) or glutamate/glycine (100 μM/10 μM), for 24 h before fixation and Hoechst staining for quantification of cell death. For all experiments, an untreated control and an SNP- or glutamate/glycine-only control was used to compare cell death and neuroprotection. Prior to treatment, the plating medium was removed and replaced with serum free medium containing 25 mM potassium chloride to prevent any potential protective effects of the serum.

#### 4.3.1. Treatment with 5K Apoptotic Medium

CGNs were co-treated with either carnosic acid or rosmarinic acid and 5K apoptotic medium. Prior to treatment, the plating medium was aspirated, and the cells were washed once with 5K apoptotic medium to remove any leftover serum. 5K apoptotic medium contains Basal Medium Eagle’s supplemented with 5 mM potassium chloride, 2 mM l-glutamate, and 2 mM penicillin-streptomycin (100 U/mL/100 µg/mL). After the first wash with 5K, the medium was removed and replaced with 1 mL of 5K apoptotic medium, which was left in each well. Wells in which the plating medium was never replaced, and wells containing 5K apoptotic medium alone were used as controls. In the remaining experimental wells, the appropriate concentration of rosmarinic acid or carnosic acid was added and the wells were left in an incubator at 3 °C for 24 h before fixation and staining with Hoechst for quantification of apoptosis.

#### 4.3.2. Protocol for Treatment with Inhibitors

CGNs were co-treated with varying concentrations of carnosic acid, and then either 5K apoptotic medium alone or containing 10 µM PD98059, 100 nM wortmannin, or 10 μM AKT Inhibitor XIII. Wells in which the plating medium was never replaced, and wells containing 5K apoptotic medium alone were used as controls. Wells containing 5K apoptotic medium plus the wortmannin, PD98059, and AKT Inhibitor XIII without carnosic acid were also included as controls. Cells were treated and then left in an incubator for 24 h before fixation, Hoechst staining, and quantification of apoptosis.

### 4.4. Fixation, Hoechst Staining, and Immunocytochemistry

Following treatment, CGNs were washed once with phosphate buffered saline (PBS; pH = 7.4) and fixed for one hour at room temperature in 4% paraformaldehyde. Cells were then washed again with PBS and stained with Hoechst at a concentration of 10 µg/mL. After washing with PBS, the cells were imaged using a Zeiss Axiovert-200M epi-fluorescence microscope. Five images were taken per well to assess apoptosis, with either duplicate or triplicate wells per experiment. Cells were counted and scored as either living or apoptotic based on nuclear morphology; CGNs having condensed or fragmented nuclei were counted as apoptotic. In some cases, the microtubule network was visualized by staining with an antibody to β-tubulin and a fluorescein isothiocyanate (FITC)-conjugated secondary antibody.

### 4.5. MTT Cell Viability Assay

As an alternative means of assessing cell death, some experiments were evaluated using an MTT viability assay. MTT (3-(4,5-dimethylthiazol-2-yl)-2,5-diphenyltetrazolium bromide) is a tetrazolium dye which is reduced by NAD(P)H-dependent cellular oxidoreductase enzymes, primarily within the mitochondria of viable cells, to yield an insoluble formazan derivative which can be solubilized and assayed colorimetrically as an indicator of cell viability. MTT data presented were obtained from four wells per treatment.

### 4.6. Data Analysis

Each experiment was performed using either duplicate or triplicate wells for each treatment, with each experiment being performed four independent times. Data are presented as the means ± SEM of the total number of experiments. Data was analyzed using a one-way ANOVA with a post hoc Tukey’s test. A *p* value of <0.05 was considered statistically significant.
